# Older adults and the disparity in oral health status; the problem and innovative ways to address it

**DOI:** 10.1186/s13584-020-00381-6

**Published:** 2020-05-04

**Authors:** Elaine O. C. Cardoso, Howard C. Tenenbaum

**Affiliations:** 1 Faculty of Dentistry, 124 Edward St. 4th floor, Toronto, ON M5G 1G6 Canada; 2 Mount Sinai Hospital, Centre for Advanced Dental Research and Care, Department of Dentistry, 600 University Avenue, Toronto, Ontario M5G 1X5 Canada

**Keywords:** Oral health, Older adults, Universal care, Progressive universalism, Preventive dental care

## Abstract

The impact of oral health inequalities on one’s ability to maintain good oral health is cumulative throughout life and accentuated in older age groups. While studies on factors influencing the decisions made by elders to seek dental care have been conducted in Israel and worldwide, the issue of access to and provision of dental care is complex. However, the need to address oral health issues is being voiced in high-level international meetings and there was never a better momentum to rethink the current oral health care delivery model beyond issues related solely to accessibility. Here we outline unique opportunities to ensure sustainable models of preventive services and oral health the effects of which would be amplified in concert with increases in the availability universal dental healthcare.

## Understanding the gap

Despite their well-known negative effects on the general population, oral diseases have been neglected historically in health policy spheres [1]. The current interventionist and treatment-oriented oral healthcare model has failed to address the needs of large proportions of the population for reasons related to general inequity and cost, particularly in the lower income and elderly populations. Therefore, a systemic reform rather than expansion of outdated oral health services has been advocated to promote more equitable access to oral care, otherwise oral health status will continue to be unaffected meaningfully [2].

The chronic and progressive nature of oral diseases throughout life [3] is so closely related to socioeconomic inequalities that a likely causal relationship between socioeconomic status and oral health is being considered [4]. Indeed, there are direct and negative effects of low socioeconomic status on functions of the cells that regulate innate immunity (polymorphonuclear neutrophils) thereby increasing risks for oral diseases, particularly periodontal diseases and possibly caries [5, 6]. This is particularly so amongst the most disadvantaged and socially marginalized aging poor, whose quality of life and general health is affected by a disproportionate burden of pain and impaired function on essential daily functions such as eating, speaking and socializing [7]. As the proportion of the population retaining natural teeth into old age is increasing, new patterns of oral disease and oral health needs are expected in a population with health-related awareness [8]. Under those circumstances, a global call for universal health coverage is in place along with increasing recognition with regard to understanding the underlying causes of oral diseases to tackle the persistent inequities in oral health.

A recent paper by Dr. Shosh Shahrabani entitled, “Factors affecting oral examinations and dental treatments among older adults in Israel” [9] points out interesting factors that influence older adults in Israel in deciding whether to undergo routine dental care. The study aims to understand the reasons why the ability to maintain good oral health is compromised as people age and enter the geriatric population. In her literature review she highlighted the importance of routine dental visits for preventing oral conditions, improving quality of life and reducing health care-related costs, and uniquely incorporated the common risk factor approach to the association between oral health and general health.

As she presents results from various studies in Israel and abroad, she discusses the issues around compliance with dental treatment specifically in the older adult population. More importantly, some key public health concepts such as the social gradient and social determinants of health permeate the discussion. Accordingly, she presents scientific evidence regarding issues of oral health inequalities throughout the life span in disadvantaged cohorts and their potential impact on individuals’ decisions to seek dental care. Equally important is the discussion on how social determinants of health drive attitudes, beliefs and oral health-related behaviours that ultimately will lead to oral conditions.

All the above being considered, the author questioned the government’s decision to include only children and people aged 75 and over in the Israeli National Health Insurance Law despite the observed burden of oral conditions on one’s quality of life and the impact of dental treatment on households’ budgets. Her sample comprised individuals between the ages of 50–75 as she hypothesized that age along with socio- economic status and health beliefs would have an impact on the individual’s decision to undergo routine dental checkups. This is a highly important and relevant topic, particularly considering the unequal use of dental services among older people in Israel despite its high rate of dentists per capita. Taking into consideration Dr. Shahrabani’s results, the author argues that accessibility to dental care under the National Health Insurance program should be extended to people younger than 75, whose dental needs are not being met due to insufficient provisions for dental checkups.

## Do traditional models of dental care funding and delivery address the gap?

As oral health status in the world has not improved in the last 25 years [10], it is time to rethink the current oral healthcare system models for oral health improvement. The drive for universal oral health coverage that is championed in the paper is very timely and echoes globally the need to address some of the deficiencies in oral health care systems currently observed worldwide [2]. This issue is particularly pertinent with the growing international momentum in which oral diseases were included in the 2011 political declaration of the United Nations (UN) High-level Meeting on Non-Communicable Diseases with a special attention to sugar and other common risk factors, its strategic alignment with the UN Sustainable Development Goals (SDG), and the 2019 UN High-level meeting on universal health coverage [2]. As the burden of oral diseases amongst the aged is already perceived as a significant public health problem in developed countries [7], critical and significant changes in dental care delivery systems are needed. While these movements present unique opportunities to ensure that sustainable models of preventive services and oral health care are developed, it is argued that the provision of more ‘universal’ oral healthcare still might not address underlying disparities in actual oral health. In fact, even with universal health coverage, there could be a widening of oral health inequalities [11] because services might still be underused among the most disadvantaged. This has been shown by others [12]; and it indicates the importance of improving both coverage and access to care.

Some additional strategies have been proposed, such as:

- Integration of oral health approaches with those for other non-communicable diseases to address issues related to sugar consumption, tobacco and alcohol use and other common risk factors to oral and systemic chronic conditions [13]. As noted earlier, these concepts have been addressed in the UN health agenda and a former executive director of the FDI World Dental Federation was recently appointed to the Board of Directors of the Non-Communicable Diseases Alliance which focuses on chronic conditions that largely affect older adults [14];
- Progressive universalism: should a country desire to implement a universal (dental) healthcare system, its focus must be on policies and strategies that address coverage without compromising equity by balancing existing health inequalities into the system framework. In other words, it must ensure that health investments are allocated where they are needed most and for those who don’t have access to affordable quality services so that the impact is mostly felt among people facing the worst inequalities.
- Profound reform in oral health care funding and delivery leading to more effective strategies for oral disease management is necessary. While the importance of the current dentist-centered care is not in question, the role and responsibilities of the dental profession in fighting the challenge of oral diseases have been questioned. Historically, dentists have been largely trained and have been practicing under the biomedical and reductionist approach in which treatment of the disease is favored over its prevention. Moreover, the fee-for-service-based payment system that is accepted commonly by individual dentists is seen as an additional incentive to high-technology and intervention-centered treatments due to their high cost. On the other hand, cost-effective interventions, including preventive treatments are disregarded in some cases even brought into discredit. Therefore, access to dental care would be extended immensely should primary care providers, including allied health providers and community-oriented oral health professionals be involved actively in preventive oral care strategies, along with health outcomes-based payment systems.

## Consequences of the gap - poor Oral health in the geriatric population

### Oral implications

There are many consequences of poor oral health in the geriatric population. From an oral health perspective alone, older individuals are prone to the development of root caries and recurrent caries, both of which can be painful and/or lead to the development of more serious dentoalveolar abscess formation. In addition, poor oral health in this population is associated with poor oral hygiene leading to oral dysbiosis: an imbalance in the oral microbiological environment that favors the buildup of pathogenic bacterial species that contribute greatly to the development of caries and periodontal/gingival inflammation. We refer to these conditions under the umbrella term “oral inflammatory load” (OIL) [15]. Oral inflammatory conditions like this can be painful, lead to tooth loss, and put the patient at risk for the development of periodontal abscesses. Taken together these problems can lead to reduced oral health related quality of life [16].

### Systemic implications

There is a groundswell of data showing that there are correlations between increased OIL, caries and tooth loss and the prevalence and severity of type 2 diabetes among other conditions (e.g. upper respiratory diseases, COPD, cardiovascular diseases, and even dementia) [17]. In analyzing health insurance databases, it has been demonstrated clearly, especially in patients with type-2 diabetes, that total healthcare costs can be reduced when conservative treatments that include regular dental cleanings are included in benefits. The overall annual cost reductions approximate $1800–$1600.00 (USD) per patient [17]. It has also been demonstrated that poor oral health in geriatric patients admitted to an acute hospital setting is a risk factor for increased mortality (> 2-fold) [18].

### What needs to be done

Clearly, based on the paper written by Dr. Shahrabani, the geriatric population in Israel is in need of dental treatment services. Yet, identification of disease in this population is hindered by the fact that among many hurdles they face, it has been difficult just to bring in such patients for assessment and treatment. If examination is restricted to the point of being impossible, there’s not much that can be done without the use of sedation. Presuming the latter is possible this could also mean that such patients would still be suitable for treatment that is *gentle, painless and minimally invasive*.

This treatment should concentrate predominantly on the prevention of disease and reestablishment of oral homeostasis including arresting caries development (recurrent or de novo) as well as prevention of periodontal inflammatory diseases (all related to oral dysbiosis); the latter leading to elevation in oral inflammatory load [15].

Considering that geriatric patients do not present for examination, and understanding why as outlined in Dr. Shahrabani’s paper, it can be proposed that such patients could be more accepting of and interested in preventive care, at a significantly earlier age than set earlier, that is *gentle, painless, minimally invasive and as reasonably priced as possible* (whether covered by a government plan, private insurance or out of pocket).

## Interventional preventive dental care

We have adopted a concept that we have called ‘Interventional Preventive Dental Care’. By approaching prevention from an *interventional* point of view we can, as much as possible, dissociate low levels of cooperativity or interest regarding oral hygiene practices from the provision of preventive therapy.

## Reduction in risks for the development of root caries and periodontal inflammation

Therefore, it is suggested here, at the very least in Israel, that a new focus be developed on the provision of proven therapeutics that can reduce dental disease load, OIL, and oral dysbiosis. This would also include treatments that can be used to arrest dental disease as well as periodontal inflammatory diseases. We propose that a relatively novel treatment be considered in patients who have demonstrable oral disease associated with elevated biofilm levels. This involves the use of a high concentration chlorhexidine coating (Prevora®), which has been shown to not only reduce the risk for caries significantly [19] but as we’ve observed also leads to *dramatic* and long lasting improvements in gingival disease and inflammation. This is effective and appreciated greatly by patients who suffer from xerostomia (Sjögren’s disease, age-related, radiation therapy related and drug related). The application of this agent is painless and given some degree of cooperation by the patient is delivered easily. Initially, four treatments (about 1 week apart) are required and then after this, treatment is generally only needed two times per year. Fortunately, there is no dental staining associated with this therapy.

For patients who have developed root caries or recurrent caries, but who would either have to undergo endodontic treatment or possibly even have to have a tooth removed due to ‘unrestorability’, the availability of sodium diamine fluoride (SDF) has created a completely new paradigm for reversal of caries, in this case for geriatric patients, although its use has been focused predominantly on pediatric patients [20]. Notably, the use of SDF will turn caries black limiting its use in the front of the mouth. However, it can certainly be used on the back teeth. Moreover, if one is dealing with patients who might be cognitively impaired, the use of SDF to reverse caries and prevent pain as well as the need for more invasive therapy even on an anterior tooth or teeth can be a boon to their care with little to no concern about the negative aesthetic effects. We have also observed that in patients treated with SDF (on more than 2–3 teeth), there are also notable reductions in gingival inflammation. Sealing of deep crevices on molar teeth using certain easily applied resins, can also prevent the development of caries irrespective of patient compliance with oral hygiene regimens. However, these resins might be difficult to apply in uncooperative patients. Topical fluoride treatments can be used more frequently and are suitable for application within the clinic setting and in some cases in the patient’s home or office. Currently, it is not recommended that fluoride treatment be the sole component for interventional preventive care. Furthermore, great care must be taken in the geriatric population when administering fluorides which can potentiate the effects of anticoagulants. Other approaches to care insofar as regulating OIL is concerned can include the use of systemic medications in the form of subantimicrobial dose doxycycline [21] with a high degree of safety (even for long term use) in order to normalize the production of matrix metalloproteinases and other proteases and cytokines implicated in the tissue destruction caused by periodontal diseases. Recognizing that, from many perspectives, reduced flow of saliva is an extremely important issue in the geriatric population, it is also recommended that patients take advantage of xylitol tablets that can be manufactured so as to adhere to either a tooth or mucosal surface (cheek) overnight, thereby stimulating salivary flow [22].

## Conclusions

Dr. Shahrabani’s paper demonstrates clearly an urgent need to develop approaches that can improve the provision of oral healthcare to the geriatric population and demonstrates that there are several impediments extant within the healthcare system including patient related issues that are making this difficult. We propose here the adoption of a philosophy of care that should overcome many of the impediments by the provision of painless, safe, effective, non-invasive and inexpensive care. This philosophy, interventional preventive dental care, can be an effective way to deliver oral healthcare that will lead to important and impactful improvements in the oral health of geriatric patients, irrespective of the patients’ or their caregivers’ abilities to look after the former’s needs effectively.

## Data Availability

Not Applicable, none used.

## Abbreviations

UN: United Nations
SDG: Sustainable Development Goals FDI World Dental Federation
OIL: Oral Inflammatory Load
COPD: Chronic Obstructive Pulmonary Disease
SDF: Sodium Diamine Fluoride
